# Characteristics of Secondary Malignancy Among Children With Primary Immunodeficiency Disorders in Saudi Arabia

**DOI:** 10.7759/cureus.93439

**Published:** 2025-09-28

**Authors:** Ali Alnakhli, Fahad Almanjomi, Hanan Alrasheedi, Hamza A Alghamdi, Nahla Mobarak

**Affiliations:** 1 Department of Pediatric Hematology and Oncology, Comprehensive Cancer Centre, King Fahad Medical City, Riyadh, SAU; 2 Department of Pediatrics, Division of Allergy and Immunology, King Fahad Medical City, Riyadh, SAU

**Keywords:** hematological malignancies, pediatric oncology, primary immunodeficiency disorders, saudi arabia, secondary malignancies, sepsis-related mortality

## Abstract

Background

Children with primary immunodeficiency disorders (PIDDs) are at an increased risk of developing malignancies due to impaired immune surveillance and genomic instability. In Saudi Arabia, limited data exist regarding the frequency, characteristics, and outcomes of secondary malignancies in this vulnerable population. Therefore, this study aimed to evaluate the clinical features, types of PIDD, associated secondary malignancies, treatment modalities, and outcomes among pediatric patients with PIDD treated at a tertiary care center in Saudi Arabia.

Methods

This retrospective observational cohort study included 28 pediatric patients diagnosed with both PIDD and malignancy at King Fahad Medical City, Riyadh, between January 2009 and January 2023. Clinical, demographic, and treatment-related data were collected and analyzed using descriptive statistics and Kaplan-Meier survival analysis.

Results

Among the 1,793 pediatric oncology patients reviewed, 28 (1.56%) were diagnosed with both PIDD and a secondary malignancy. Most patients were male (64.3%) and Saudi nationals (82.1%). Hematologic malignancies were predominant (85.7%), with secondary hemophagocytic lymphohistiocytosis (42.9%) and diffuse large B-cell lymphoma (21.4%) being the most common. Griscelli syndrome (32.1%) and ataxia-telangiectasia (17.9%) were the most frequently observed PIDD. Chemotherapy was administered to 96.4% of patients, with dose adjustments required in 25.9%. Febrile neutropenia was reported in 70.4%, and infections were documented in 75% of cases. Bone marrow transplantation was performed in 39.3% of patients. Progression-free survival was 92.2%, and overall survival was 64.3%, with sepsis accounting for 80% of deaths.

Conclusion

Children with PIDD are at high risk for developing early-onset, aggressive malignancies, especially hematologic cancers. Despite significant treatment-related complications and infection susceptibility, curative approaches such as chemotherapy and transplantation are feasible. Early identification, individualized treatment, and aggressive infection control are crucial to improving survival outcomes in this high-risk population.

## Introduction

Primary immunodeficiency disorders (PIDDs) refer to a diverse group of conditions characterized by impaired function in one or more components of the immune system. This impairment compromises both adaptive and innate immunity, making affected individuals more susceptible to recurrent infections, autoimmune reactions, abnormal inflammation, and an increased risk of malignancy development [1]. The International Union of Immunological Societies has phenotypically classified 485 inherited immune disorders, including 55 novel monogenic defects and one autoimmune phenocopy, highlighting the broad spectrum of associated clinical features [2]. Similarly, in another study, 330 distinct disorders, each associated with 320 different genetic defects, have been categorized as PIDD [3].

The prevalence of PIDD varies across populations and is significantly influenced by the degree of consanguinity within the population, indicating autosomal recessive (AR) as a predominant pattern of inheritance. PIDDs are more common in the Middle East and North Africa (MENA) region, where consanguineous marriages are common, with an overall incidence ranging from 20% to 50% [4]. PIDDs are typically grouped into three primary groups: (1) antibody deficiencies, related to the B-cell or humoral immunodeficiencies primarily affecting antibody production; (2) cell-mediated immunodeficiencies, involving impaired cellular effector mechanisms while antibody levels remain normal; and (3) combined immunodeficiencies, which involve both humoral and cellular defects [5].

Cancer immune surveillance is a key mechanism in the immune system responsible for detecting and eliminating developing tumors without external therapeutic interventions [6,7].

In individuals with PIDD, impaired DNA repair processes lead to genomic instability, thereby increasing the risk of developing cancers such as B- or T-cell lymphoma, Hodgkin lymphoma, leukemia, carcinoma, medulloblastoma, and neuroblastoma [8,9].

Children and adolescents who are diagnosed with PIDD typically develop malignancies at younger ages, with a higher prevalence among males [10]. The incidence in affected individuals is 10,000 times higher than in the general population, with a 4-25% higher likelihood of malignancy in children diagnosed with PIDD [4,7,11,12].

Malignancy development is the second most common cause of mortality in patients with PIDD following infectious complications. The increased cancer risk in this population is attributed to weakened immune surveillance systems and exposure to certain specific viruses [4,11,13].

The advancement in diagnostics, supportive care, and therapeutic strategies has extended the lifespan of patients with both primary and secondary immunodeficiencies [9].

However, effective management of PIDD remains challenging due to the rarity of these disorders, which considerably limits scientific advancements related to the treatment and prevention of associated malignancies [13].

Comprehensive data on the frequency of secondary malignancies among children diagnosed with PIDDs in Saudi Arabia are lacking. Therefore, this study aimed to determine the most prevalent types of secondary malignancies, identify associated immune deficiencies, assess the overall incidence of malignancies, and characterize disease-specific features in this population. To our knowledge, this represents one of the first systematic analyses of PIDD-associated malignancies in Saudi Arabia, thereby addressing a critical gap in regional data. The findings have important clinical implications, as they may inform local guidelines, support the development of national registries, and guide early screening strategies to improve timely diagnosis and tailored management for this vulnerable group.

This study was presented on April 1, 2025, at the SIOP Asia 2025 conference, and the corresponding abstract appeared in the OncoDaily Medical Journal.

## Materials and methods

Study design

This retrospective observational cohort study was conducted in the Department of Pediatric Hematology and Oncology, King Fahad Medical City (KFMC), Riyadh, Saudi Arabia. The study was designed to evaluate the clinical characteristics, treatment modalities, and outcomes of pediatric patients with PIDD who subsequently developed secondary malignancies. Medical records of eligible patients treated between January 2009 and January 2023 were reviewed.

The study protocol was reviewed and approved by the Institutional Review Board (IRB) of KFMC (approval number: 23-196; IRB registration number with OHRP/NIH, USA: IRB00010471), and all relevant ethical guidelines and regulations were followed.

Patients and sample size

A total of 28 out of 1,793 pediatric oncology patients were identified and included in the final analysis. Given the rarity of the condition and the retrospective nature of the study, all eligible patients within the specified timeframe were enrolled.

Data collection

Clinical, demographic, and treatment-related data were obtained from the institutional electronic medical records and patient charts. Data included age at diagnosis, sex, nationality, type of PIDD, type of malignancy, treatment received (surgery, chemotherapy, radiotherapy, bone marrow transplantation (BMT)), treatment modifications, complications (e.g., febrile neutropenia, documented infections), relapse, and survival outcomes.

Inclusion criteria

This study included children and adolescents younger than 14 years of age who were diagnosed with PIDD and subsequently developed secondary malignancies. Secondary malignancies were defined as cancers occurring as a consequence of the underlying immunodeficiency rather than as therapy-related malignancies. Both male and female patients were eligible for inclusion, regardless of nationality.

Exclusion criteria

Patients older than 14 years at the time of malignancy diagnosis were excluded from the study to maintain the pediatric focus of the research.

Statistical analysis

All data obtained were entered and analyzed using IBM SPSS Statistics for Windows, Version 26.0 (Released 2019; IBM Corp., Armonk, New York, United States). Descriptive statistics were used to summarize demographic and clinical characteristics of the study population. These included frequencies, percentages, means, and standard deviations for categorical and continuous variables, respectively.

Kaplan-Meier survival analysis was conducted to estimate overall survival (OS) among the study population. OS was defined as the time from diagnosis of malignancy to either death or last follow-up. Patients alive at the last follow-up were censored. The survival probabilities were plotted as Kaplan-Meier curves.

Due to the relatively small sample size (n=28), no formal power analysis was conducted. However, all eligible patients within the specified study period were included to maximize statistical power. Missing data were reported and accounted for in the analysis accordingly.

## Results

Demographics and patient characteristics

A total of 28 pediatric patients diagnosed with PIDDs who subsequently developed secondary malignancies were included in the study. The cohort consisted predominantly of males, with 18 male patients (64.3%) and 10 female patients (35.7%), corresponding to a male-to-female ratio of 9:5. Most patients were Saudi nationals (82.1%; n=23), while 17.9% (n=5) were non-Saudi.

Most patients (39.3%; n=11) presented with symptoms during infancy or before the age of two years. Ten patients (35.7%) presented between two and 10 years of age, while seven patients (25%) were older than 10 years at the time of disease presentation. In 25% of the cases, malignancy was the initial presenting symptom, suggesting that in most patients, malignancy was the first clinical indicator of underlying PIDD. All clinical, diagnostic, therapeutic, and outcome data are summarized in Table 1.

**Table 1:** Clinical, diagnostic, therapeutic, and outcome profiles of pediatric oncology patients with PIDDs Retrospective single-center pediatric cohort (N=28). Categorical variables are presented as n (%). †Hematologic toxicity and febrile neutropenia are calculated among patients who received chemotherapy (n=27). ‡Documented infection categories are among patients with ≥1 confirmed infection (n=21). §BMT donor/source percentages are among transplanted patients (n=11). "Malignancy as first presentation" indicates a cancer diagnosis preceding the diagnosis of PIDD. "Chemotherapy modified" refers to a dose reduction implemented in response to treatment-related toxicity or the patient's clinical condition. Toxicities were graded using standard CTCAE criteria (version per Methods). "CNS involvement" refers to primary CNS tumors and/or CNS disease. Survival reflects status at the last follow-up. "Progression-free survival" indicates the absence of clinical/radiologic progression by the last follow-up (equivalently, progression events occurred in 7.1%). Values are rounded to one decimal; minor discrepancies may occur due to rounding. This table provides descriptive statistics only; no between-group hypothesis tests are presented here. ADA2: adenosine deaminase 2; AT/RT: atypical teratoid/rhabdoid tumor; BMT: bone marrow transplant; CNS: central nervous system; CTCAE: Common Terminology Criteria for Adverse Events; DLBCL: diffuse large B-cell lymphoma; HLH: hemophagocytic lymphohistiocytosis; IL2RG: interleukin-2 receptor gamma; ITK: IL-2-inducible T-cell kinase; MALT1: mucosa-associated lymphoid tissue lymphoma translocation protein 1; PIDD: primary immunodeficiency disorder

Category	Subcategory	Percentage (%)
Sex	Female	35.7
	Male	64.3
Nationality	Non-Saudi	17.9
	Saudi	82.1
Age at presentation	<2 years (infancy)	39.3
	2-10 years	35.7
	>10 years	25
Malignancy as first presentation	Yes	25
	No	75
Primary immunodeficiency disorder	Griscelli syndrome	32.1
	Ataxia-telangiectasia	17.9
	DiGeorge syndrome (22q11.2 deletion)	7.1
	Chediak-Higashi syndrome	7.1
	Others (each): MALT1, ITK, IL2RG, ADA2, etc.	3.6 (each)
Malignancy types	Hematological	85.7
	CNS tumors	10.7
	Solid tumors	3.6
Specific malignancies	Secondary HLH	42.9
	DLBCL	21.4
	Hodgkin lymphoma	10.7
	Pineoblastoma, synovial sarcoma, AT/RT, etc.	3.6-7.1 (each)
CNS involvement	Yes	35.7
	No	64.3
Surgery performed	Yes	35.7
	No	64.3
Radiation therapy	Yes	10.7
	No	89.3
Chemotherapy given	Yes	96.4
	No	3.6
Modification of chemotherapy	Modified	25.9
	Not modified	74.1
Hematological toxicity (graded)	Grade III	81.5
	Grade II	14.8
	Grade I	3.7
Febrile neutropenia	Yes	70.4
	No	29.6
Documented infections	Bacterial+viral	47.6
	Viral only	33.3
	Bacterial only	9.5
	Fungal only	4.8
	Mixed (bacterial+viral+fungal)	4.8
BMT	Yes	39.3
	No	60.7
BMT donor type	Matched related donor	45.5
	Haploidentical allogeneic	27.3
	umbilical cord transplant	18.2
	Autologous stem cell	9.1
Relapse	Yes	7.1
	No	92.9
Progression-free survival	Yes	7.1
	No	92.9
Overall survival	Alive	64.3
	Died	35.7
Cause of death	Sepsis	80
	Progression/relapse	20

Types of PIDDs

The study population exhibited different types of PIDDs. The most common condition was Griscelli syndrome, identified in 32.1% of patients. Other notable immunodeficiencies included ataxia-telangiectasia (17.9%), DiGeorge syndrome (22q11.2 deletion; 7.1%), and Chediak-Higashi syndrome (7.1%).

**Figure 1:**
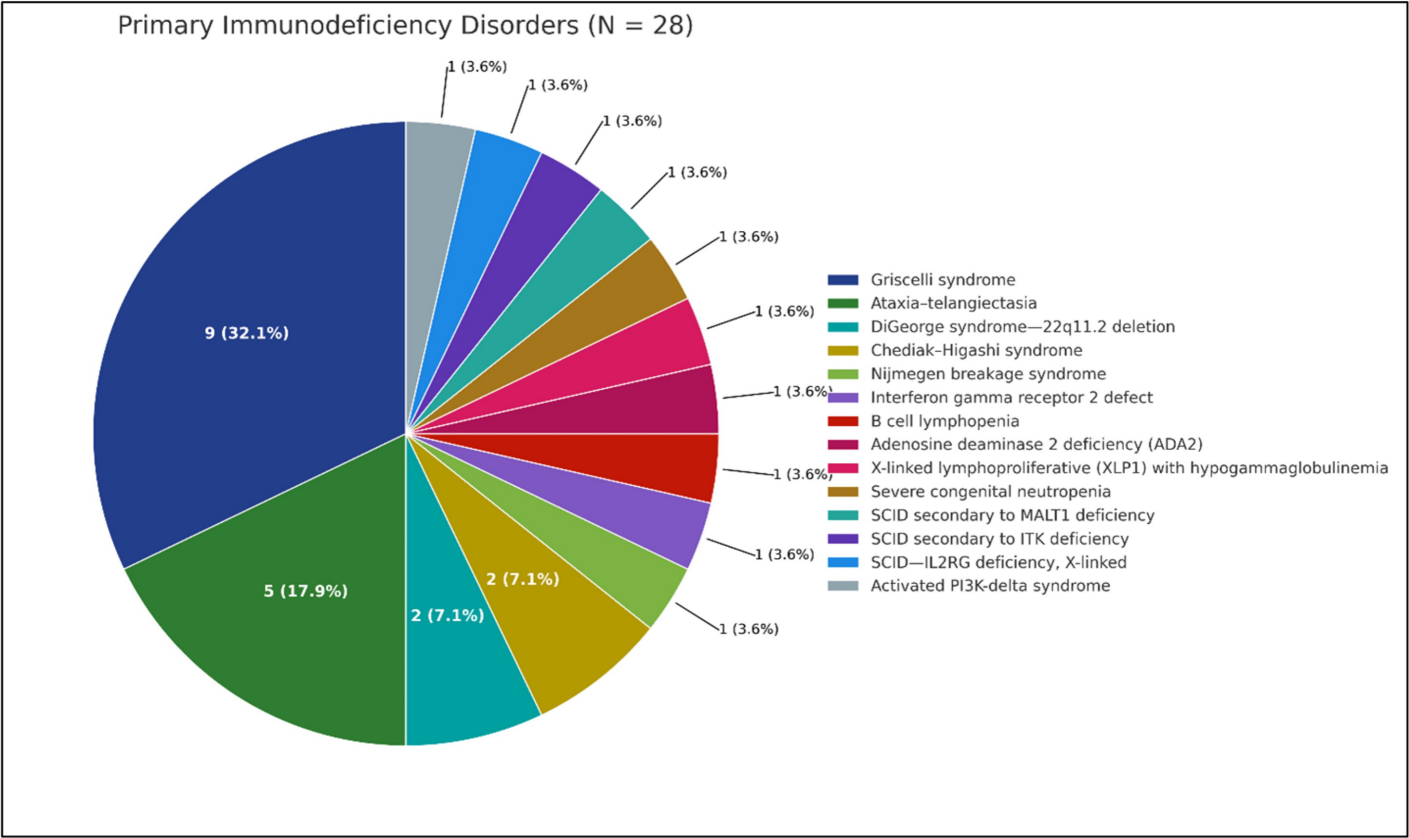
Distribution of primary immunodeficiency disorders (N=28) Pie chart with counts and percentages: Griscelli (9, 32.1%), ataxia-telangiectasia (5, 17.9%), DiGeorge/22q11.2 (2, 7.1%), Chediak-Higashi (2, 7.1%), and 10 single-patient entities (3.6% each): Nijmegen breakage syndrome; interferon-γ receptor 2 defect; B-cell lymphopenia; ADA2 deficiency; XLP1 with hypogammaglobulinemia; severe congenital neutropenia; SCID due to MALT1 deficiency; SCID due to ITK deficiency; X-linked SCID-IL2RG; and activated PI3K-δ syndrome. Percentages use N=28 as the denominator; descriptive only (no hypothesis testing). ADA2: adenosine deaminase 2; MALT1: mucosa-associated lymphoid tissue lymphoma translocation protein 1; ITK: IL-2-inducible T-cell kinase; IL2RG: interleukin-2 receptor gamma; SCID: severe combined immunodeficiency

Several patients had extremely rare immunodeficiencies, such as severe combined immunodeficiency secondary to MALT1, ITK, or IL2RG (X-linked) deficiencies, as well as Nijmegen breakage syndrome, interferon gamma receptor defects, adenosine deaminase 2 deficiency, and X-linked lymphoproliferative disorder. Each of these accounted for approximately 3.6% of the cohort (Table 1). These findings show the genetic heterogeneity and clinical complexity of PIDD in the context of malignancy development. The distribution of primary immunodeficiency disorders is shown in Figure 1.

Types of malignancy and presentation

Hematologic malignancies were predominant, occurring in 85.7% (n=24) of patients. Central nervous system (CNS) tumors were diagnosed in 10.7% (n=3), and one patient (3.6%) had a solid tumor. The most frequent specific malignancy was secondary hemophagocytic lymphohistiocytosis (HLH), present in 42.9% (n=12). Diffuse large B-cell lymphoma was identified in 21.4% (n=6), while Hodgkin lymphoma was diagnosed in 10.7% (n=3). Other less frequent malignancies included high-risk T-cell acute lymphoblastic leukemia (7.1%), pineoblastoma (7.1%), synovial sarcoma (3.6%), and atypical teratoid/rhabdoid tumor (3.6%). One patient had a dual diagnosis of Hodgkin lymphoma with secondary HLH (Table 1).

Treatment modalities and complications

CNS involvement was observed in 35.7% (n=10) of patients. Surgical intervention was required in 35.7% (n=10) of cases, while radiation therapy was administered to 10.7% (n=3) (Table 1).

**Figure 2:**
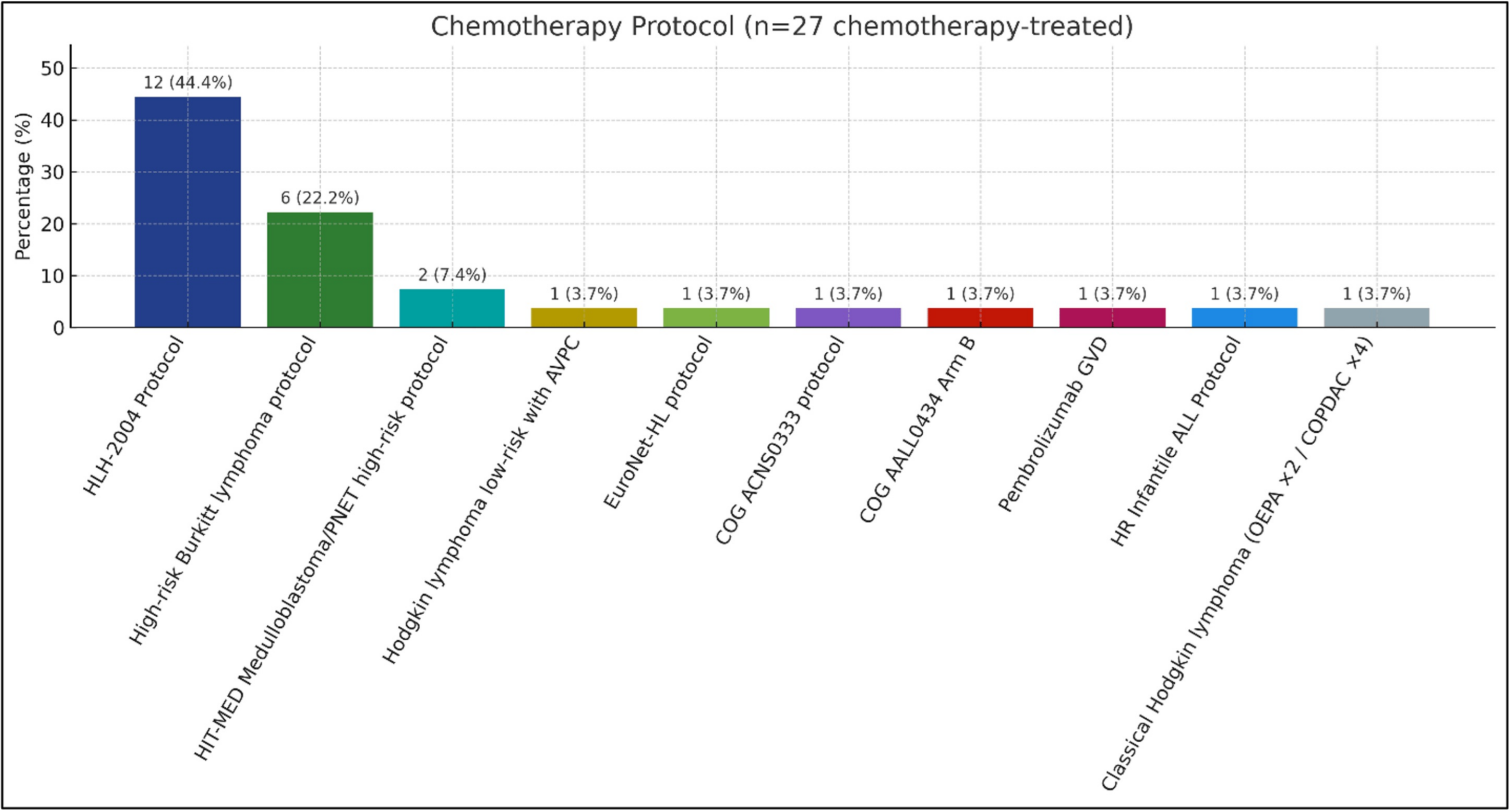
Chemotherapy protocols among chemotherapy-treated patients (n=27) Bar chart showing n (percentage) for each protocol: HLH-2004 (12, 44.4%), high-risk Burkitt (6, 22.2%), HIT-MED medulloblastoma/PNET high-risk (2, 7.4%), and single-patient protocols (1, 3.7%) each: Hodgkin low-risk with AVPC; EuroNet-HL; COG ACNS0333; COG AALL0434 Arm B; pembrolizumab GVD; HR infantile ALL; and classical Hodgkin (OEPA×2/COPDAC×4). Percentages use n=27 as the denominator; descriptive only (no hypothesis testing). HLH: hemophagocytic lymphohistiocytosis; HIT-MED: Hirntumoren (German brain tumors)-medulloblastoma, ependymoma, diverse; PNET: primitive neuroectodermal tumor; AVPC: doxorubicin, vincristine, prednisone, and cyclophosphamide; EuroNet-HL: EuroNet Hodgkin lymphoma; COG: Children's Oncology Group; GVD: gemcitabine, vinorelbine, and liposomal doxorubicin; HR infantile ALL: high-risk infantile acute lymphoblastic leukemia; OEPA: vincristine, etoposide, doxorubicin, and prednisone; COPDAC: cyclophosphamide, vincristine, dacarbazine, and prednisone

Chemotherapy was the primary treatment modality, received by 96.4% (n=27) of patients. Notably, 25.9% (n=7) had chemotherapy modifications due to drug toxicity, highlighting the susceptibility of this patient population to treatment-related adverse effects (Table 1). The distribution of chemotherapy protocols is shown in Figure 2.

Chemotherapy-related hematological toxicity was a major clinical complication. Grade III toxicity was most common, affecting 81.5% (n=22), followed by Grade II in 14.8% (n=4) and Grade I in 3.7% (n=1) (Table 1).

**Figure 3:**
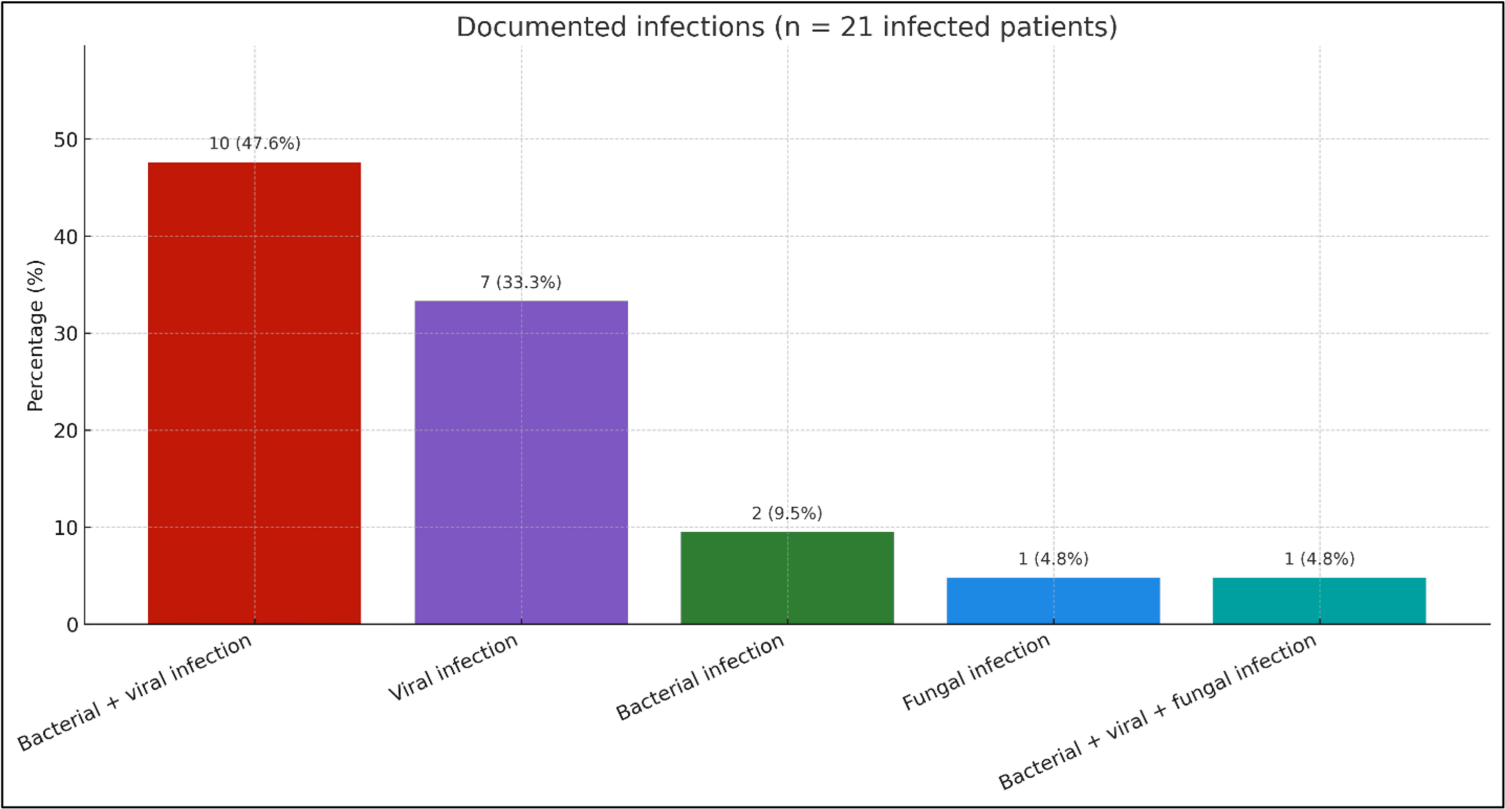
Documented infections among patients with ≥1 infection (n=21) Bar chart showing n (percentage) for infection categories: bacterial+viral (10, 47.6%), viral only (7, 33.3%), bacterial only (2, 9.5%), fungal only (1, 4.8%), and bacterial+viral+fungal (1, 4.8%). Percentages use n=21 as the denominator; descriptive only (no hypothesis testing).

Febrile neutropenia was reported in 70.4% (n=19) of patients, representing a major clinical concern during treatment. Among patients who developed infections (n=21), the most common infection type was a combination of bacterial and viral pathogens (47.6%; n=10), followed by isolated viral infections (33.3%; n=7), isolated bacterial infections (9.5%; n=2), isolated fungal infections (4.8%; n=1), and mixed bacterial, viral, and fungal infections (4.8%; n=1). Microbiological analysis revealed that *Klebsiella pneumoniae* was the most frequently isolated bacterial organism, accounting for 28.6% of all bacterial isolates, followed by *Enterobacter cloacae* and *Mycobacterium tuberculosis* (each 14.3%). In the viral category, Epstein-Barr virus was the most frequently detected virus, identified in 56.3% of viral infections, followed by cytomegalovirus (25%). Fungal infections were rare, with only *Aspergillus* and *Candida sake *each detected in one patient (Table 1). The distribution of documented infections is shown in Figure 3.

BMT

**Figure 4:**
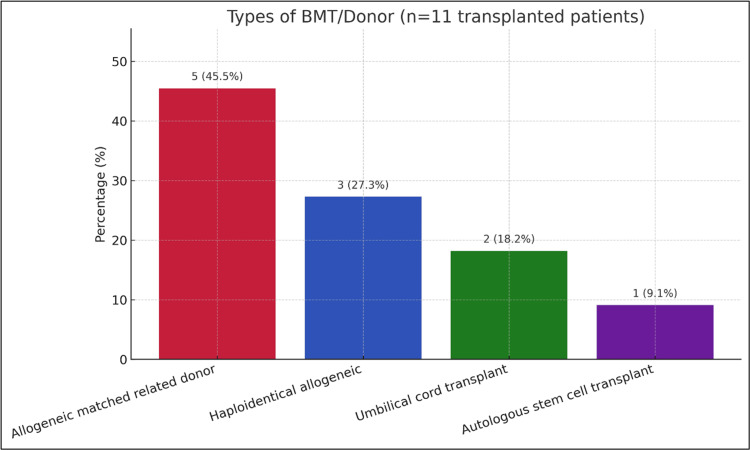
Types of BMT/donor among transplanted patients (n=11) Bar chart showing counts and percentages among transplanted patients: matched related donor (5, 45.5%), haploidentical allogeneic (3, 27.3%), umbilical cord (2, 18.2%), and autologous stem cell (1, 9.1%). Percentages use n=11 as denominator; descriptive only (no hypothesis testing). Minor rounding may occur. BMT: bone marrow transplant

BMT was performed in 39.3% of the patients (n=11). Among these, 45.5% (n=5) received grafts from matched related donors. Haploidentical allogeneic transplants were used in 27.3% (n=3), umbilical cord blood transplants in 18.2% (n=2), and autologous stem cell transplants in 9.1% (n=1) (Table 1). These transplant strategies were chosen based on disease status, donor availability, and institutional protocols. The distribution of transplant donor/source types is shown in Figure 4.

Relapses and survival outcomes

**Figure 5:**
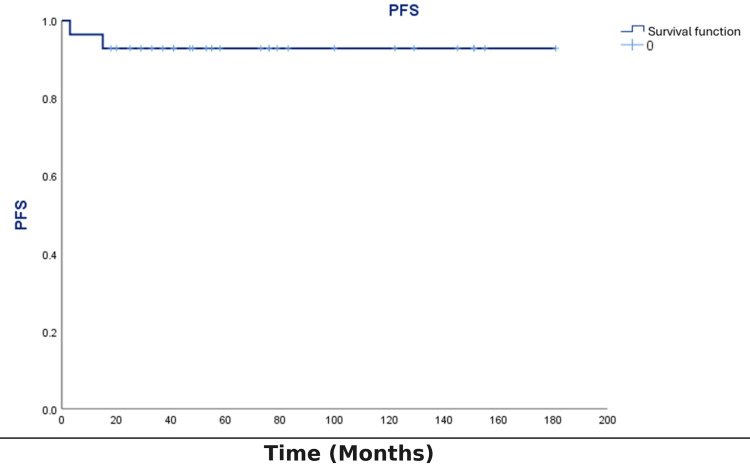
PFS in pediatric oncology patients with primary immunodeficiency disorders (N=28) Kaplan-Meier estimate of PFS; time in months from malignancy diagnosis to first progression/relapse or death. Event: progression/relapse or death. Censoring: event-free at last contact (tick marks shown). Step function; descriptive only (no hypothesis testing). PFS: progression-free survival

**Figure 6:**
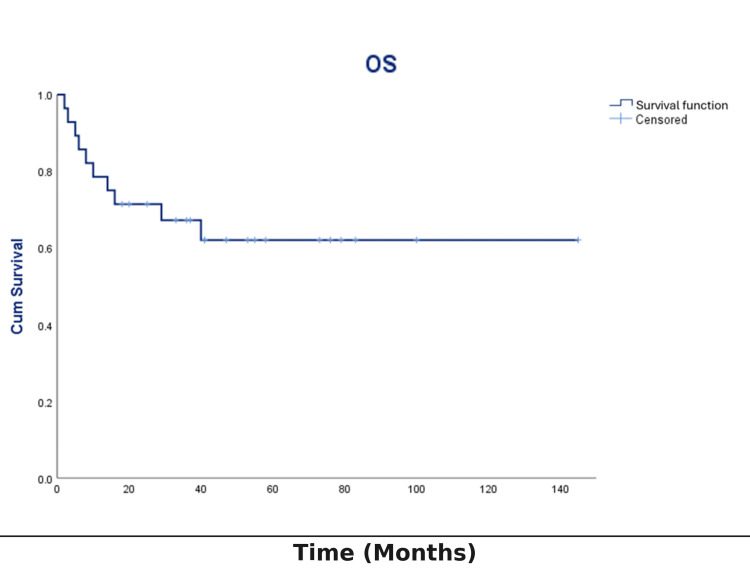
OS in pediatric oncology patients with primary immunodeficiency disorders (N=28) Kaplan-Meier estimate of OS; time in months from malignancy diagnosis to death from any cause. Event: death. Censoring: alive at last contact (tick marks shown). Step function; no hypothesis testing or confidence bands displayed. OS: overall survival

Relapse occurred in two patients (7.1%). The progression-free survival rate was 92.2% (Figure 5). OS was 64.3% (18/28; Figure 6). Ten patients (35.7%) died during the study period. The primary cause of death was sepsis (80%), followed by disease progression or relapse (20%) (Table 1).

Kaplan-Meier survival analysis

Kaplan-Meier survival analysis revealed that OS steadily declined over time, with the most substantial decrease observed during the initial years following diagnosis. The survival curve showed no early plateau, indicating continued risk of mortality throughout the follow-up. Patients alive at the end of the study were censored. The probability of survival at various times reflected the severity of both the underlying immunodeficiency and the associated malignancy.

## Discussion

This study presented a detailed analysis of pediatric patients with PIDD who developed secondary malignancies and were treated at a tertiary care center in Saudi Arabia. Among the 1,793 pediatric oncology patients treated at our center over a 14-year period, 28 were diagnosed with both PIDD and malignancy. A male predominance (64.3%) was observed, consistent with existing studies, where X-linked immunodeficiencies such as X-linked agammaglobulinemia, common variable immunodeficiency, and ataxia-telangiectasia predominantly affected males, leading to a higher incidence of malignancies [14,15].

Most patients were Saudi nationals (82.1%), possibly due to the high rates of consanguinity in the country, which are known to increase the incidence of AR immunodeficiencies [16].

More than one-third of patients exhibited disease symptoms before the age of two years, highlighting that malignancy can emerge very early in the clinical course of PIDD. This finding is consistent with that of previous studies, which indicate that malignancies can manifest shortly after the diagnosis of PIDD, often within a year [14]. Notably, in 25% of patients, the malignancy itself was the first presenting feature.

Griscelli syndrome and ataxia-telangiectasia were the most frequent PIDDs identified, followed by DiGeorge syndrome and Chediak-Higashi syndrome. This diversity shows the genetic heterogeneity of PIDDs in Saudi Arabia. As expected, hematological malignancies were predominant (85.7%). These findings are consistent with those of a study conducted at the Children's Hospital of Brescia, which focused on the incidence of tumors among patients with PIDD. Most tumors, comprising 66.7% (22/33), were hematological in nature [14]. Another study highlighted the strong association between PIDDs and hematologic malignancies, indicating that patients with these immunodeficiencies face a significantly higher risk of developing hematological cancers [17].

Most patients (96.4%) underwent chemotherapy, while surgical and radiation therapies were applied more selectively. Treatment-related toxicity was notable: over one-quarter of patients required chemotherapy dose modifications due to toxicity, and 81.5% experienced Grade III hematological toxicity. Febrile neutropenia was documented in over 70% of cases, and 75% had documented infections, often polymicrobial in nature. These findings are consistent with those of previous studies showing that children with PIDD have impaired tolerance to standard oncologic therapies and are highly susceptible to opportunistic infections. A study emphasized that patients with PIDD often present with advanced disease and have a higher risk of complications from standard treatments, such as chemotherapy, due to their underlying immune deficiencies [15]. These complications not only compromise the quality of life and increase the healthcare burden but also require dose reductions or delays, potentially affecting cancer outcomes. Our findings underscore the importance of individualized therapy, intensive supportive care, and early antimicrobial interventions.

BMT was performed in 39.3% of patients using a variety of donor sources. Matched related donors were preferred when available; however, alternative donors, including haploidentical and umbilical cord sources, were also utilized. This reflects the growing use of alternative donor transplantation strategies in immunocompromised patients. The relatively low relapse rate (7.1%) and moderate OS (64.3%) suggest that curative therapies are feasible in this population despite the high-risk context. Sepsis was the leading cause of death (80%), indicating that infection control is as critical as oncologic management in patients with PIDD. Other studies have also shown that sepsis is the leading cause of death in patients with hematological malignancies, with mortality rates reaching up to 60% [18]. In pediatric oncology, approximately 24-26% of deaths are associated with sepsis, predominantly in patients with leukemia [19]. A study observed that severe sepsis in critically ill children with cancer showed a mortality rate of 34% [20]. Early recognition, aggressive management of infections, and prophylactic strategies must remain integral to treatment protocols.

Our study confirms that malignancy in pediatric patients with PIDD is a complex, multifactorial phenomenon, often characterized by early onset, hematologic predominance, high treatment-related morbidity, and variable survival. The primary limitation of this study is its retrospective nature and small sample size. Additionally, genetic confirmation was not uniformly available for all cases, which may limit the precision of immunodeficiency classification. Despite these limitations, the study provides essential baseline data and highlights several trends relevant to clinical practice.

Future research should prioritize establishing national and regional registries for children with PIDD and cancer to allow for larger, multicenter studies. Early genetic testing, personalized treatment regimens, and integration of novel immunotherapeutic strategies may improve both oncologic and survival outcomes in this vulnerable patient group.

Limitations of the study

The primary limitation of this study is its retrospective nature and small sample size. Additionally, genetic confirmation was not uniformly available for all cases, which may limit the precision of immunodeficiency classification. Despite these limitations, the study provides essential baseline data and highlights several trends relevant to clinical practice.

## Conclusions

This single-center retrospective study, conducted at a tertiary care center in Saudi Arabia, provides important insights into the clinical presentation, treatment strategies, and outcomes of pediatric patients with PIDD who developed secondary malignancies. Hematological malignancies were the predominant cancer type, often presenting at a young age and associated with considerable treatment-related morbidity. Sepsis emerged as the leading cause of mortality, highlighting the critical need for comprehensive supportive care. To our knowledge, this is one of the first systematic analyses of PIDD-associated malignancies in Saudi Arabia, contributing to both regional and global literature. While the findings are valuable, they should be interpreted in light of the small sample size and single-center design, which limit generalizability. Moving forward, establishing national registries and conducting multicenter collaborative studies will be essential to validate these results and guide the development of personalized treatment strategies to improve outcomes and quality of life in this vulnerable population.
